# Honghua extract mediated potent inhibition of COVID-19 host cell pathways

**DOI:** 10.1038/s41598-022-15338-9

**Published:** 2022-08-22

**Authors:** Malika Madikyzy, Meruyert Tilegen, Guldan Nazarbek, Chenglin Mu, Aidana Kutzhanova, Xugang Li, Cuiping Ma, Yingqiu Xie

**Affiliations:** 1 Department of Biology, School of Sciences and Humanities, Nazarbayev University, 53 Kabanbay Batyr Ave, 010000 Nur-Sultan, Republic of Kazakhstan; 2 Sino-German Joint Research Center on Agricultural Biology, State Key Laboratory of Crop Biology, College of Life Sciences, Shandong Agricultural University, Tai’an, 271018 China; 3 Shandong Provincial Key Laboratory of Biochemical Engineering, Qingdao Nucleic Acid Rapid Detection Engineering Research Center, College of Marine Science and Biological Engineering, Qingdao University of Science and Technology, Qingdao, 266042 China; 4 Present Address: Zhejiang University-Hangzhou Global Scientific and Technological Innovation Center, Hangzhou, China

**Keywords:** Biochemistry, Drug discovery

## Abstract

Honghua (*Carthami flos*) and Xihonghua (*Croci stigma*) have been used in anti-COVID-19 as Traditional Chinese Medicine, but the mechanism is unclear. In this study, we applied network pharmacology by analysis of active compounds and compound-targets networks, enzyme kinetics assay, signaling pathway analysis and investigated the potential mechanisms of anti-COVID-19. We found that both herbs act on signaling including kinases, response to inflammation and virus. Moreover, crocin likely has an antiviral effect due to its high affinity towards the human ACE2 receptor by simulation. The extract of Honghua and Xihonghua exhibited nanozyme/herbzyme activity of alkaline phosphatase, with distinct fluorescence. Thus, our data suggest the great potential of Honghua in the development of anti-COVID-19 agents.

## Introduction

Nanotechnology has been applied to Traditional Chinese Medicine including drug delivery, nanozyme, and herbzyme^1–3^. However, more natural product-based nanozyme, herbzyme in nanoscale extracts of Traditional Chinese Medicine should be investigated. Honghua herb, *Carthami flos,* based on Latin pharmacological nomenclature, was used for the treatment of various diseases including angina pectoris, hypertension, coronary heart disease, etc^4^. Those effects probably resulted from different curing mechanisms such as protective effect on myocardium and brain tissues, antithrombotic and pain-relieving effects^4^. Similarly, Xihonghua herb, *Croci stigma* or saffron, has been widely used in the treatment of fever, asthma, bronchitis, atherosclerosis, depression and anxiety^5,6^. The stigma of Xihonghua herb contains volatile and nonvolatile compounds, such as crocin, safranal, picrocrocin and kaempferol, which have antiviral, antioxidant, anti-inflammatory, and immunostimulating effects^7,8^. Molecular dynamics simulation study showed that crocetin, one of the main compounds of Xihonghua, has a high binding affinity to the spike (S) protein and protease of SARS-CoV-2, which makes it a promising agent for COVID-19 treatment^9^.

It is obvious that the COVID-19 pandemic affected almost every aspect of human lives around the world. COVID-19 can affect more severely on people who suffer from various chronic diseases such as diabetes mellitus, disorders in heart function and elevated blood pressure^10^. Effective treatment for COVID-19 is extremely important to reduce mortality rate. Kinase elevation is reported as a COVID-19 characteristic of host cell response to induce phosphorylation events which can be targets for the treatment^11,12^. Thus, targeting the kinase in anti-COVID-19 by natural products or enzymes which inhibit kinases would be a promising avenue as we recently suggested^13^. Studies suggest that Traditional Chinese Medicine plays a role in anti-COVID-19^14^^.^ An assessment is needed to overcome the difficulty of the sophisticated chemical compound of the herbs entry to the drug targets. Few studies investigated the potent nanoscale particles for the delivery of anti-COVID-19 medicine with nanozyme^1^ or herbzyme activities^3^. Therefore, to evaluate the potential of nanoscale Honghua in anti-COVID-19, network pharmacology with additional analyses of nanozyme from extracts were performed for exploring the potent mechanisms of Honghua and Xihonghua targeting host cell pathways.

## Materials and methods

### Decoction, scanning electron microscope (SEM) investigation and fluorescence spectrum analysis

We claim here that the experimental research here which does not include field studies on plants of either cultivated or wild, which includes the collection of plant materials, was in compliance with relevant institutional, national, and international guidelines and legislation. As commercial natural products packaged but not as wild field plants, the dried Honghua and Xihonghua stigma were purchased from local pharmacy shops in China and UAE respectively. The decoction of dried Honghua and Xihonghua stigmas were performed by boiling in hot ddH2O at 100 °C followed by filtration with a 200 nm filter (ThermoFisher). During the period of decoction, pellets were removed and the particles in the solution were collected. The samples were put to coverslip for drying until applied to SEM analysis for particles morphology and size measurement. Finally, the samples were measured by a fluorescence spectrometer (Agilent Cary Eclipse fluorescence spectrometer). 350 nm is used for emission and excitation wavelength and fluorescence was observed between the wavelength starts from 350 nm and stops in 800 nm. 20 nm was set up as the excitation and the emission slits. Water was used as a control. Scan rate was set up as 600 nm/min.

### Nanozyme/herbzyme activity assay

Phosphatase activity was measured using substrate of Nitro-Blue Tetrazolium (NBT)/5-bromo, 4-chloro, 3-indolylphosphate (BCIP) from ThermoFisher (1-Step™ NBT/BCIP Substrate Solution). Enzyme kinetics were measured by different substrate concentrations as indicated in the figure legend^3^.

### Active ingredients of Honghua/Xihonghua with targets

The active components of both herbs were identified using the Database TCMSP^15^. The Chinese herb names “Honghua” and “Xihonghua” were used as keywords, and the retrieved ingredients were filtered according to the oral bioavailability (OB) not less than 30% and drug-likeness (DL) not less than 0.18^16,17^. The potential targets of these compounds were obtained from TCMSP. For further analysis of nanoscale herbs, the OB and DL were not filtered. With updated database, the HERB (http://herb.ac.cn/) was also used for target gene list and analysis^18^.

### Protein–protein interaction (PPI) map

GeneCards database website online tool was used to identify the genes related to COVID-19, kinases by entering the keyword “COVID-19” ,“kinase” or alkaline phosphatase by “alkaline phosphatase”. Then Venny 2.1 website online tool^19^ and Metascape (https://metascape.org/^20^) were used to obtain the intersections of Honghua or Xihonghua gene targets with COVID-19, kinase, alkaline phosphatase related genes with following further analysis. The intersection targets were analysed by the STRING platform with Version 11.5 by website online tool^21^. The score was set to 0.7. Then the PPI network was constructed from java based tool, Cytoscape ver 3.8.2^22^.

### Gene Ontology (GO) enrichment

The official gene symbols of Honghua and Xihonghua and COVID-19 intersection targets were entered into DAVID^23^ and the p-value was set to ≤ 0.01. Then the figure drawing and graphic displaying was performed by website (www.bioinformatics.com.cn) to make a bubble dot diagram of the results. KEGG analysis (https://www.genome.jp/kegg/)^24–26^ via KOBAS database tool which additionally provides bubble dot diagrams of the results (http://kobas.cbi.pku.edu.cn)^27^ was used. Metascape^20^ was also used for cluster and GO/KEGG combined analysis.

### Molecular docking

The PubChem website was used to obtain the 3D structure of the compounds of Honghua and Xihonghua, and then SDF files were converted into Protein Data Bank (PDB) files by Open Babel 2.3.2. The structures of receptor proteins were searched on the PDB website online. Then the dehydration of the receptor proteins was performed using PyMol software^28^. AutoDock Vina 1.1.2.^29^-based molecular docking was performed via supervising by Xiamen University collaborators.

## Results

As shown in Table 1, Honghua herb contains 21 ingredients which have the required characteristics (OB ≥ 30%, DL ≥ 0.18). While only 5 ingredients of Xihonghua herb meet the criteria, 4 more compounds safranal, picrocrocin, crocin I, II, and were selected from literature review^4–8^ (Table 2).

**Table 1:** Active ingredients of Honghua.

Mol ID	Compound	Molecular formula	Molecular weight	OB (%)	DL
MOL002719	6-Hydroxynaringenin	C_15_H_12_O_6_	288.27	33.23	0.24
MOL002714	Baicalein	C_15_H_10_O_5_	270.25	33.52	0.21
MOL002698	Lupeol-palmitate	C_46_H_80_O_2_	665.26	33.98	0.32
MOL000006	Luteolin	C_15_H_10_O_6_	286.25	36.16	0.25
MOL001771	Poriferast-5-en-3beta-ol	C_29_H_50_O	414.79	36.91	0.75
MOL000358	Beta-sitosterol	C_29_H_50_O	414.79	36.91	0.75
MOL002773	Beta-carotene	C_40_H_56_	536.96	37.18	0.58
MOL000953	CLR	C_27_H_46_O	386.73	37.87	0.68
MOL002706	Phytoene	C_40_H_64_	545.04	39.56	0.50
MOL002776	Baicalin	C_21_H_18_O_11_	446.39	40.12	0.75
MOL000422	Kaempferol	C_15_H_10_O_6_	286.25	41.88	0.24
MOL002707	Phytofluene	C_40_H_62_	543.02	43.18	0.50
MOL002695	Lignan	C_25_H_30_O_8_	458.55	43.32	0.65
MOL000449	Stigmasterol	C_29_H_48_O	412.77	43.83	0.76
MOL002721	Quercetagetin	C_15_H_10_O_8_	318.25	45.01	0.31
MOL002757	7,8-dimethyl-1H-pyrimido[5,6-g]quinoxaline-2,4-dione	C_12_H_10_N_4_O_2_	242.26	45.75	0.19
MOL000098	Quercetin	C_15_H_10_O_7_	302.25	46.43	0.28
MOL002710	Pyrethrin II	C_22_H_28_O_5_	372.50	48.36	0.35
MOL002694	Kinobeon A	C_20_H_20_O_6_	356.40	48.47	0.36
MOL002680	Flavoxanthin	C_40_H_56_O_3_	584.96	60.41	0.56
MOL002712	6-Hydroxykaempferol	C_15_H_10_O_7_	302.25	62.13	0.27

**Table 2:** Active ingredients of Xihonghua.

Mol ID	Compound	Molecular formula	Molecular weight	OB (%)	DL
MOL001406	Crocetin	C_20_H_24_O_4_	328.44	35.30	0.26
MOL000422	Kaempferol	C_15_H_10_O_6_	286.25	41.88	0.24
MOL000098	Quercetin	C_15_H_10_O_7_	302.25	46.43	0.28
MOL000354	Isorhamnetin	C_16_H_12_O_7_	316.28	49.60	0.31
MOL001389	n-heptanal	C_7_H_14_O	114.19	79.74	0.59
MOL001405	Crocin I	C_44_H_64_O_24_	977.97	2.54	0.12
MOL001407	Crocin II	C_37_H_52_O_19_	800.8	1.65	0.21
MOL000720	Safranal	C_10_H_14_O	150.21	39.56	0.04
MOL001409	Picrocrocin	C_16_H_26_O_7_	330.37	33.71	0.04

**Figure 1:**
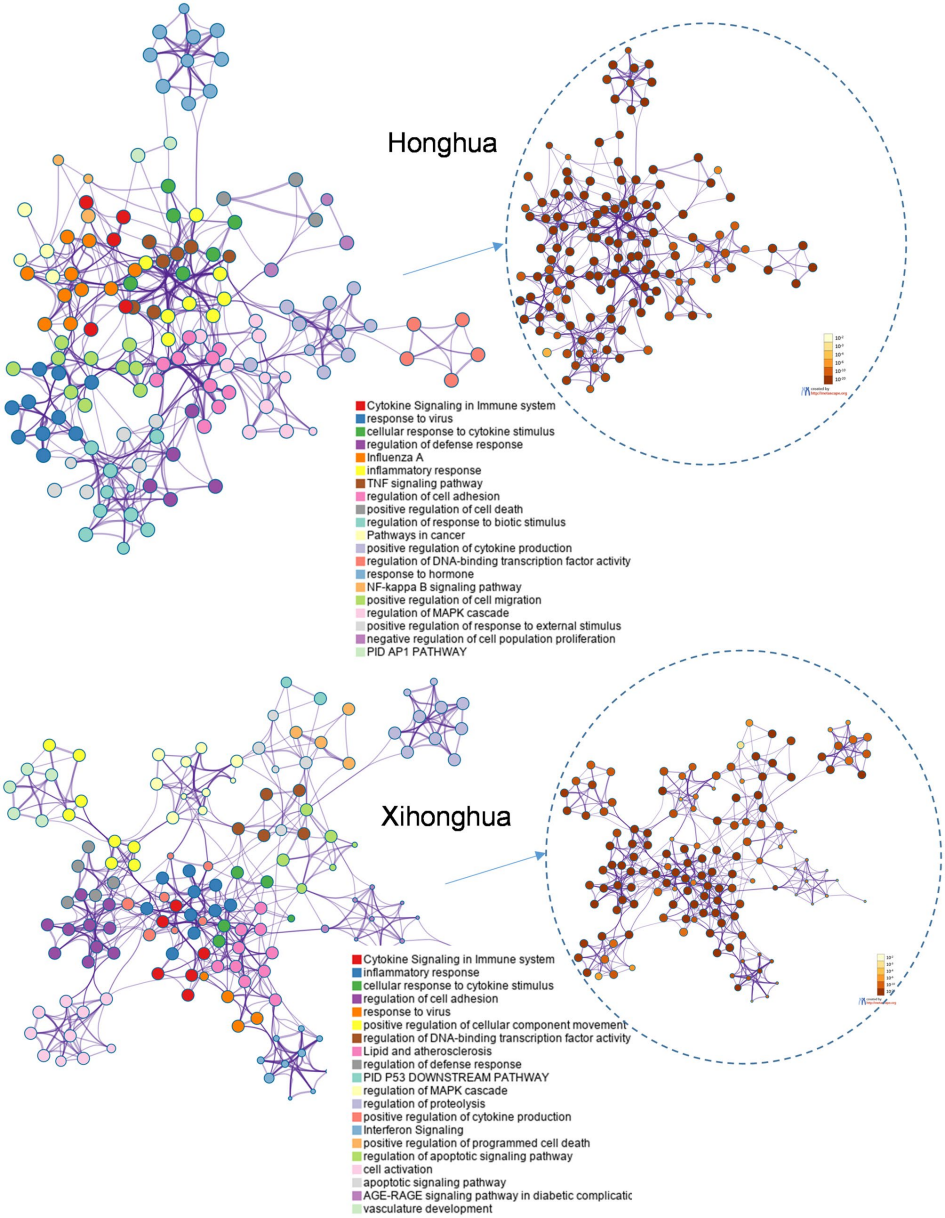
Protein–protein interaction networks of Honghua and Xihonghua related gene targets against COVID-19 targets analyzed by Metascape^20^. A subset of clusters shown by network layout with terms which are indicated by a circle node, in which size represents the number of genes, and color indicates the function or pathways. Similarity scores more than 0.3 were linked by an edge as the thickness indicating the score. The dashed inset shows the nodes by color intensity corresponding to p-value.

The intersection targets were analysed by the network of cluster by Metsascape of COVID website^20^ using updated targets of HERB (Fig. 1). In the Honghua network, responses to virus, hormone, cytokine, inflammatory, cancer pathways, transcription regulation, cell adhesion and MAPK pathway are the darker and the larger cluster nodes, which suggests the significance in anti-COVID-19 mechanism. In the Xihonghua network, in addition to cytokine response, which is similar to Honghua, responses to virus, cytokine, inflammatory, cell adhesion and MAPK pathway, differential clusters include p53 pathway, cell apoptosis, cell movement, and lipid signaling are found.

**Figure 2:**
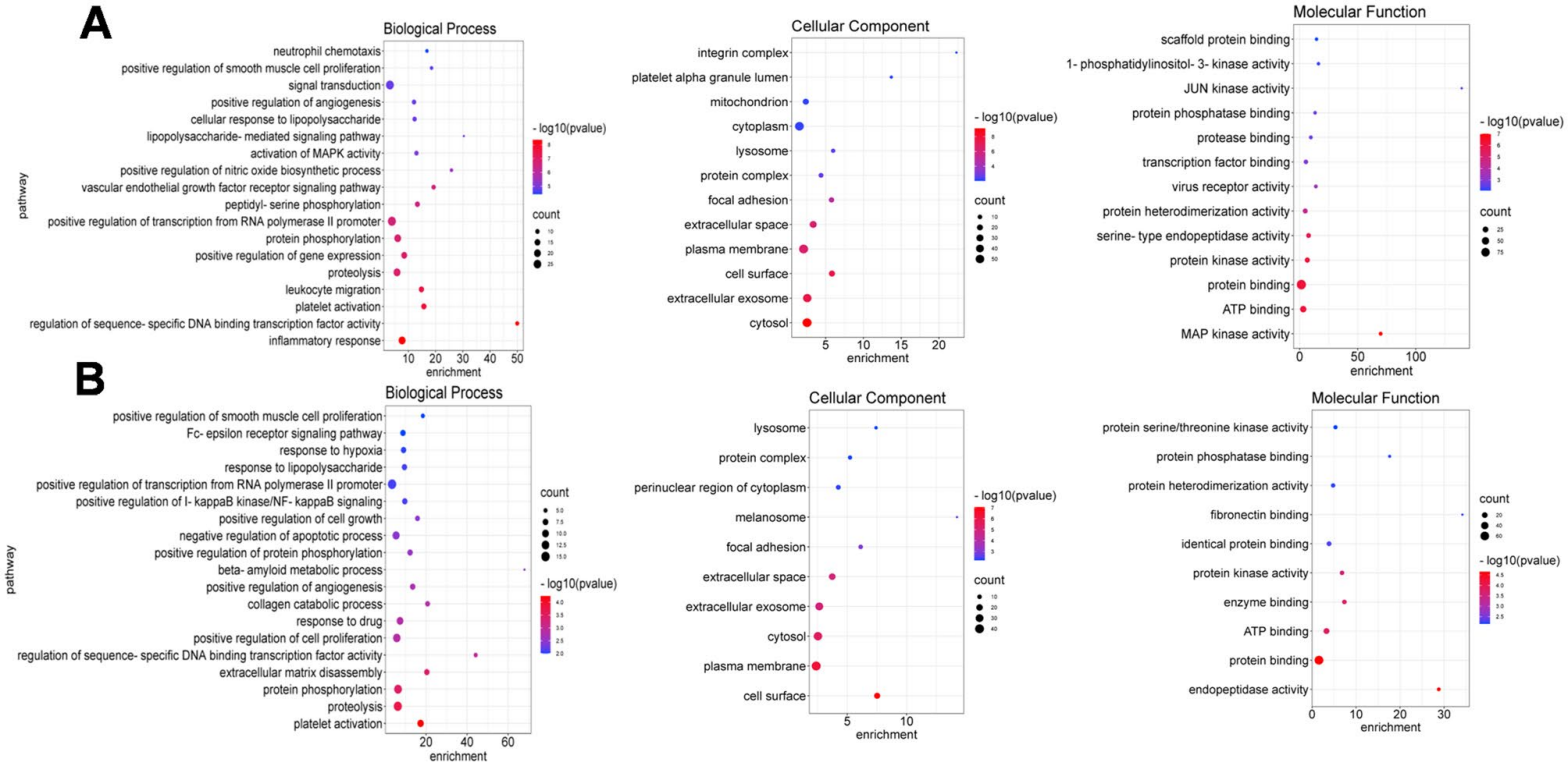
GO enrichment of Honghua (**A**) and Xihonghua (**B**) against COVID-19.

Functional enrichment analysis performed by DAVID revealed 75 significant biological processes (BP) in which the Honghua herb might participate. Inflammatory response, regulation of sequence-specific DNA binding transcription factors activity, platelet activation, leukocyte activation and proteolysis were the most enriched (Fig. 2). Xihonghua herb is also involved in similar BP, such as platelet activation, proteolysis, protein phosphorylation, as well as positive regulation of cell proliferation, response to hypoxia and response to drug (Fig. 2). The analysis also showed 20 enriched molecular functions (MF) of Honghua, including MAP kinase activity, protein kinase activity, virus receptor activity and scaffold protein binding. Whereas Xihonghua is enriched in 12 MF and is most significantly involved in protein binding, endopeptidase activity, ATP binding and fibronectin binding (Fig. 2). Finally, the Honghua herb can act on 19 cellular components (CC) and cytosol, extracellular exosomes and cell surface were the most important. Xihonghua mainly acts on the cell surface, plasma membrane, cytosol and extracellular space (Fig. 2).

**Figure 3:**
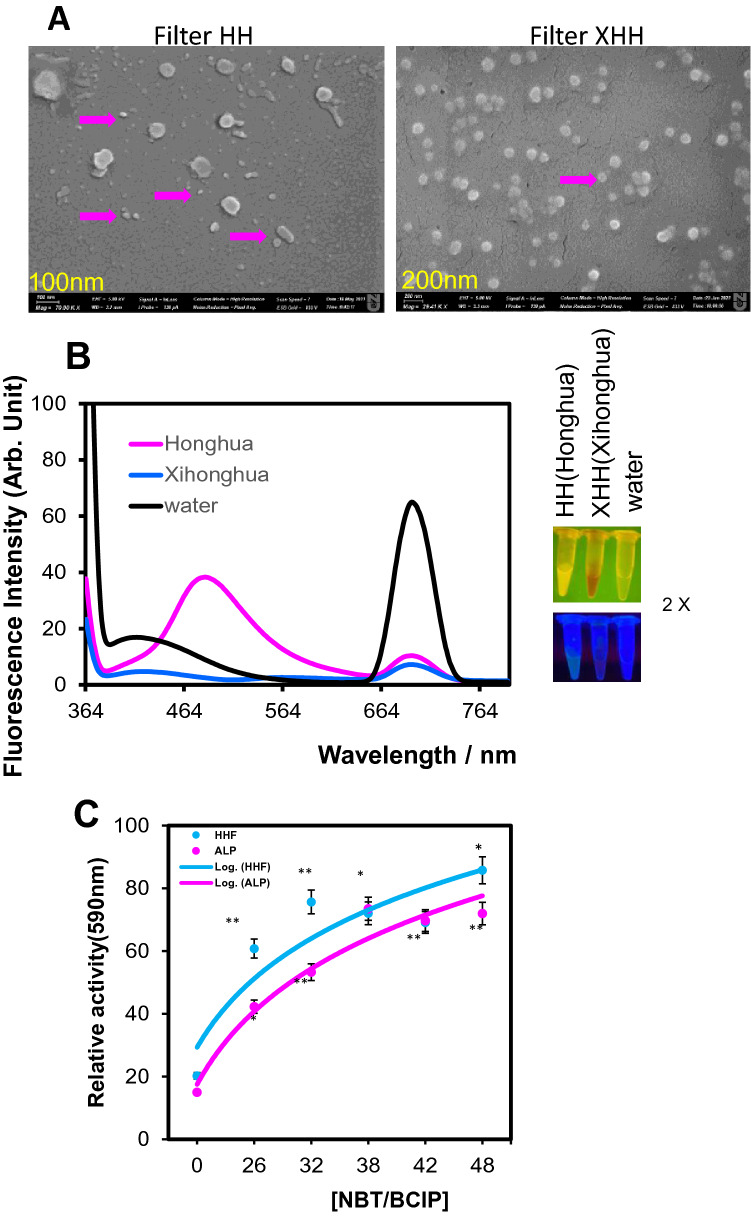
Honghua and Xihonghua extracts exhibited enzyme activities. (**A**) SEM of Honghua (HH) and Xihonghua (XHH). (**B**) The fluorescence spectrum of Honghua and Xihonghua at the nanoscale. (**C**) Nanozyme kinetics of nanoscale of filtered Honghua (HHF) extract.

Given that the nanoscale Traditional Chinese Medicine may have nanozyme/herbzyme activity and that COVID-19 has the phosphorylation events which can be targeted in the treatment, we tested the potential nanozyme activity of the two herbs. First, we made the extraction of Honghua and Xihonghua, measured the nanoscale compound complex of extract and found that the nano-sized particles exist in the extracts (Fig. 3A). The fluorescence characteristics of the nano-sized particles in Honghua are much stronger than that of Xihonghua with small-sized particles about 20–30 nm and larger particles with 100–200 nm (Fig. 3A, B). Xihonghua showed less assembly with larger sized particles.

We further tested the nanozyme potential of phosphatase. As expected, Honghua exhibits the phosphatase activity with comparable protein enzyme of Alkaline Phosphatase (ALP) in *Km* (Fig. 3C). In addition, we found that pH affects the nanozyme activity (Supplementary Figure 2). Though the pH13 showed the highest activity for both herbs, the Honghua has weaker activities at a broader pH range, while Xihonghua barely has the activity at pH other than pH13 (Supplementary Figure 2). Thus, the minor difference between the two herbs on nanozyme activity suggests that the distinct compound on the surface of the particles may also affect enzyme function.

**Figure 4:**
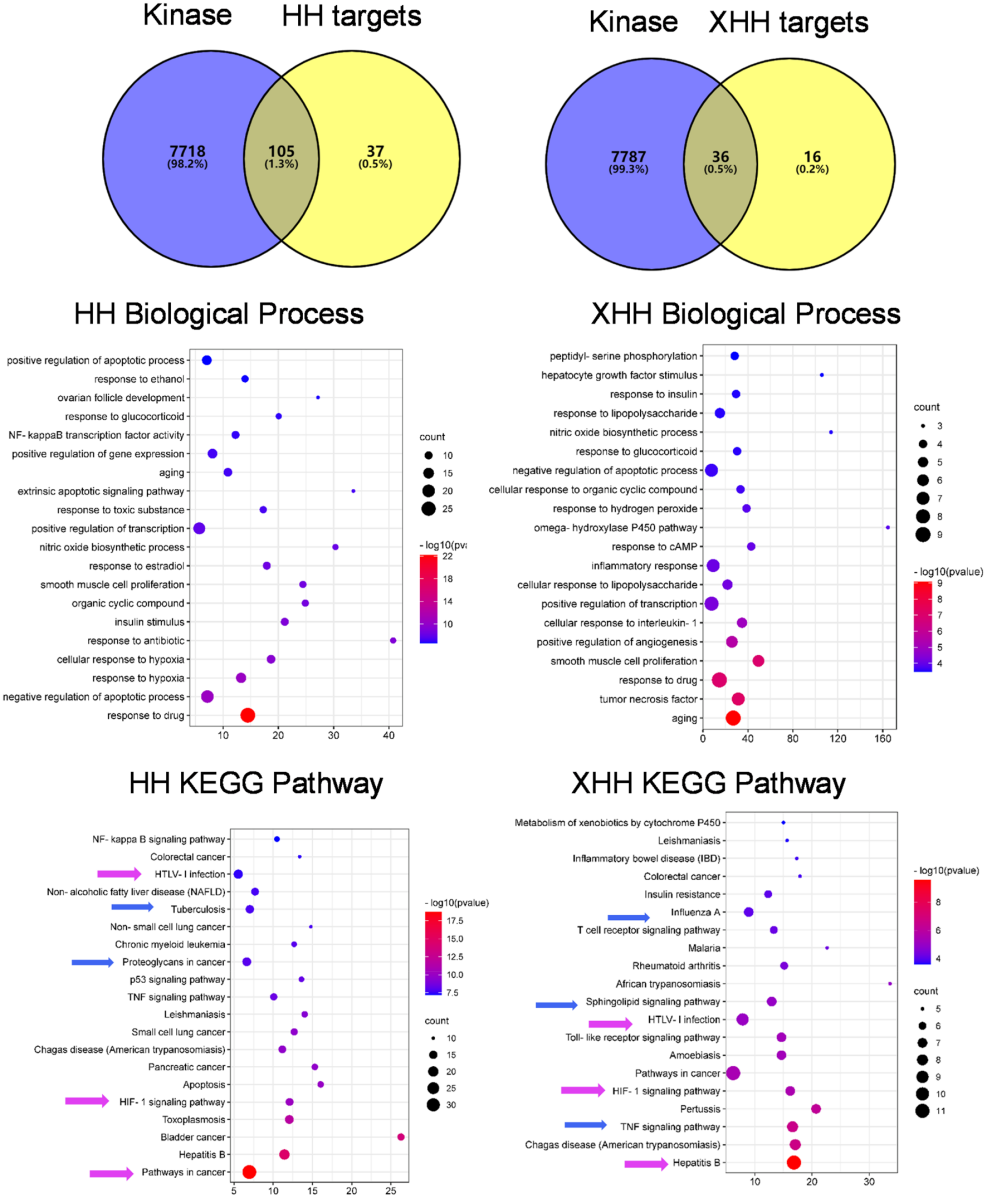
The Honghua and Xihonghua targets against kinases pathways by GO and KEGG analysis of intersections network.

**Figure 5:**
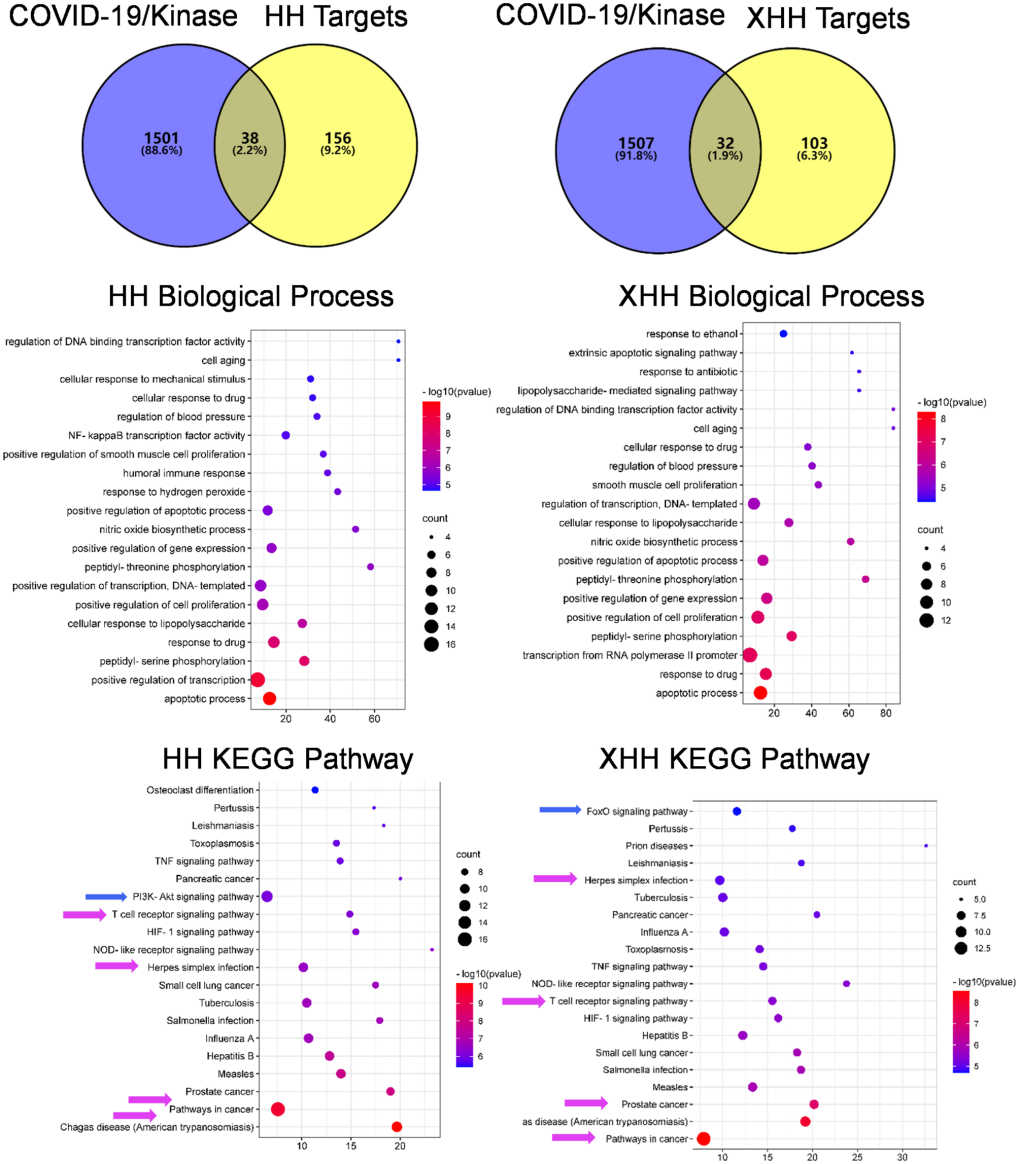
The Honghua and Xihonghua targets against COVID-19/kinases pathways by GO and KEGG analysis of intersections network. Blue arrows indicate the differential pathways while pink arrows indicate common pathways in KEGG enrichment analysis.

Next, we analyzed the potential of the two herbs against kinases by pathway analysis. We applied the intersection of all of the herb ingredients without OB, DL selection from HERB web site, and kinase-related genes. Most of the pathways, including HIF-1 signaling and HTLV-1 infection, for both of the herbs, are common, but the differences are Tuberculosis (TB) infectious disease, proteoglycans in cancer pathways in Honghua and TNF, influenza, pathways in Xihonghua (Fig. 4). Then we analyzed the two herbs with the COVID-19/kinase intersection genes, and we found that the differential pathways are PI3K-AKT in Honghua and FOXO in Xihonghua, while the additional common pathways are HPV infection, T cell receptor, cancer, prostate cancer, and lung cancer pathways (Fig. 5). Thus, though both Honghua and Xihonghua exhibit similar mechanisms against COVID-19 by targeting kinases, cytokine, and inflammatory response, there might be some pathways specific to each herb.

Finally, molecular docking was carried out to predict the most favorable binding modes of the Xihonghua and Honghua ingredients with one of the SARS-CoV-2 targets, human ACE2 (hACE2). The docking scores of safranal, picrocrocin and crocin are − 5.7, − 7.0 and − 9.2 kcal/mol respectively, while lopinavir and remdesivir were used as controls^30^.

The crocin molecule may form hydrogen bonds with the receptor protein hACE2 Gln101, Asn194, Tyr196, Glu208, His401, Asp350 amino acid residues, and hydrophobic interaction with amino acid residues His195, Gln102, Gly205, Lys562, Ala348, Tyr385, Arg393, Phe40, he39, Trp69, Ala99, Gln98, Leu391, Leu85, Asn103 (Table 3). The amino acid residues Gln102, Gln98, Leu95 may form hydrogen bonds with the picrocrocin molecule, while Trp203, Tyr202, Asp206, Gly205, Ala99, Lys562 amino acid residues may form a hydrophobic interaction. The binding mode between the safranal molecule and the receptor protein hACE2 predicted that the amino acid residue Gln98 and safranal molecule may form hydrogen bond, while amino acid residues Leu95, Lys562, Glu564, Pro565, Trp566, Val209, Asn210, Glu208 form hydrophobic interactions.

**Table 3:** Potent binding of compound to hACE2.

Compound	Hydrogen bonds	Hydrophobic interactions
Crocin	Gln101	His195
	Asn19	Gln102
	Tyr196	Gly205
	Glu208	Lys562
	His401	Ala348
	Asp350	Tyr385
		Arg393
		Phe40
		Phe39
		Trp69
		Ala99
		Gln98
		Leu391
		Leu85
		Asn103
Picrocrocin	Gln102	Trp203
	Gln98	Tyr202
	Leu95	Asp206
		Gly205
		Ala99
		Lys562
Safranal	Gln98	Leu95
		Lys562
		Glu564
		Pro565
		Trp566
		Val209
		Asn210
		Glu208

**Figure 6:**
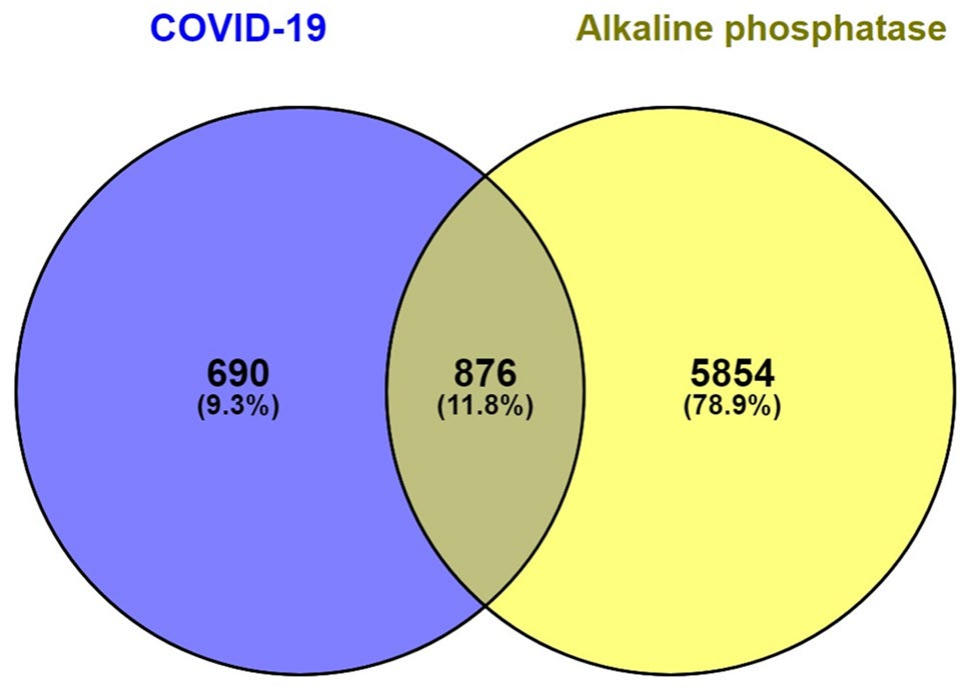
Intersectional potent signaling analysis of alkaline phosphatase related genes list against COVID-19 by Venn Diagram^19^.

Since the phosphatase characteristics of Honghua may involve in the drug delivery at the nanoscale, we further tested the phosphatase mediated signaling in anti-COVID-19 pathways. We used the intersection of alkaline phosphatase related genes and COVID-19 related genes (Fig. 6) to perform GO and KEGG analysis by Metascape^20^ (report from https://metascape.org/COVID/). The most significant pathways were found to be the cytokine storm, phosphorylation, and some interesting pathways are infection and inflammation (report from https://metascape.org/COVID/).

**Figure 7:**
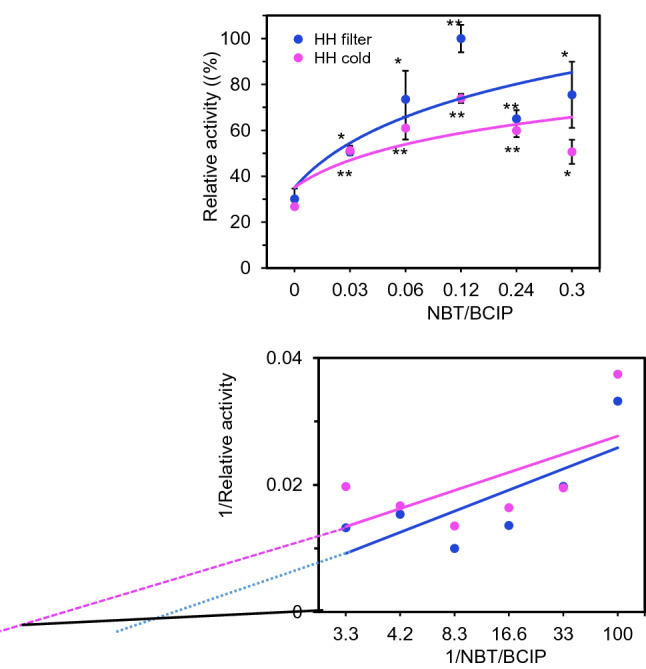
Alkaline phosphatase nanozyme activity of extract of Honghua (HH filter) catalyzing NBT/BCIP compared to the cold water extract. *p < 0.05; **p < 0.01.

Given the possible phosphatase activity of the Honghua nanozyme by nanoparticle assembly when boiled, we tested the activity of cold water extract and boiling water extract in catalysis of substrate NBT/BCIP. We found the phosphatase activity by boiling indeed has higher kinetics, suggesting the nanoparticles may not increase the binding affinity but may increase the enzyme reaction rate (Fig. 7).

## Discussion

We reported both large molecular compound-based network and nanoscale phosphatase analysis of two similar Chinese herbal medicines in relation to anti-COVID-19. Most importantly, we found that the nanozyme activity of phosphatase might contribute to the COVID-19/kinase intersection networks. At the potential nanoscale, high OB and DL might be neglected with nanoscale effect on both surface compound and nanozyme activity. Both herbs reveal the phosphatase-kinase inhibition pathways, as well as the immune response, AKT kinases, cancer-like signaling, and transcriptional regulation pathways, which may show a potential therapeutical benefit against COVID-19.

Database screening showed that the herbs have some active ingredients in common when DL value is greater than or equal to 0.18% and OB at least 30%. These compounds are kaempferol and quercetin, suggesting the two herbs may have similar function in anti-COVID-19 which is consistent with our further analysis in GO, and KEGG.

COVID-19 patients showed the elevated alkaline phosphatase even associated with high risk of death of COVID-19 related or liver injury^31,32^. Phosphatase elevation can be a biomarker associated event, and the nanoparticles may enhance the phosphatase marker which can be used to indicate the marker spatiality as we also found in carbon dots nanoparticles^33^. In addition, the high pH related phosphatase activity may be modified by doping with other element or ions to enhance the enzyme activity under normal physiological condition, which will warrant the potent pharmacological application for treatment or imaging as a marker.

hACE2 as the host cell receptor of SARS-CoV-2 entry, plays essential roles in anti-COVID-19. Molecular docking showed that safranal, picrocrocin and crocin molecules found in the composition of Xihonghua might have binding modes with hACE2 protein. Crocin has a higher docking score which can be used as a basis for new drug development against COVID-19. Among the already investigated pharmacological effects of crocin, considerable anti-inflammatory was found in database, in particular, anti-inflammatory effects on respiratory organs of the body are of significancer which can be used to treat inflammation of the respiratory system caused by COVID-19. Those effects were observed in mice administered with inflammation of airways caused by allergy^34^. Moreover, crocin possesses cardioprotective effects due to its ability to regulate oxidative stress^35^. Since COVID-19 affects heart function, crocin may also be used for prevention of COVID-19 associated heart attack^36^. In addition, even the docking score of safranal was lower than that of other compound, it indicates an antidiabetic protective impact on rats with diabetes induced by streptozotocin via enforcing antioxidant mechanism^37^. Thus, safranal might be still considerable as a potential agent in antidiabetic associated with COVID-19^37^. While ACE2 active site binding to its substrate is through Arg 273, the other catalysis involving sites such as His345, His505 also play roles^38^. During intermediate status of catalysis, His345 can form hydrogen bonds with substrate but His505 can not^38^. Thus, based on the simulation, there might be no direct interaction with active site binding of compounds we investigated. More experimental testing would warrant the inhibition efficiency.

Recently, many novel designs of anti-COVID-19 drugs, especially nanomedicine have been reported. Nanoceria, which has catalase enzyme activity, could be used for anti-COVID-19 to disrupt MAPK kinase signaling^39^. This report suggest the potential enzyme-based targeting of cell signaling of host cells for anti-COVID-19. Natural product bilirubin nanomedicine can inhibit inflammatory response which has potential in anti-COVID-19^40^. Based on our data that Honghua or Xihonghua may target cell adhesion, which may link to extracellular matrix based targeting in nanomedicine^41^. Moreover, carbon dots could also be applied in anti-COVID-19^42,43^. In addition to nanomedicine, as a matter of fact, nanoparticles such as lipid nanoparticles have been used in delivery of mRNA for COVID-19 vaccines^44^. Thus, applying nanotechnology in natural products and traditional medicine would be a novel avenue to reduce side effects and enhance the drug delivery efficacy.

## Conclusion

We report that both large molecular compounds and nanoscale nanozyme of Honghua and Xihonghua may inhibit COVID-19 signaling by kinase and other pathways. While Honghua and Xihonghua nanoparticles may exhibit a nanozyme/herbzyme activity of phosphatase and crocin showed the potent binding to ACE2, the potential of Honghua and Xihonghua in anti-COVID-19 should be considered to develop natural product and enzyme-based inhibitions of SARS-CoV-2 entry.

## Supplementary Information

Supplementary Information.
