# Supplementary material for: Honghua extract mediated potent inhibition of COVID-19 host cell pathways
Source: Sci Rep. 2022 Aug 22;12:14296. doi: 10.1038/s41598-022-15338-9 (PMC9395372; doi:10.1038/s41598-022-15338-9)
Supplement: Supplementary file 1 — Supplementary Information. [file 41598_2022_15338_MOESM1_ESM.pdf]

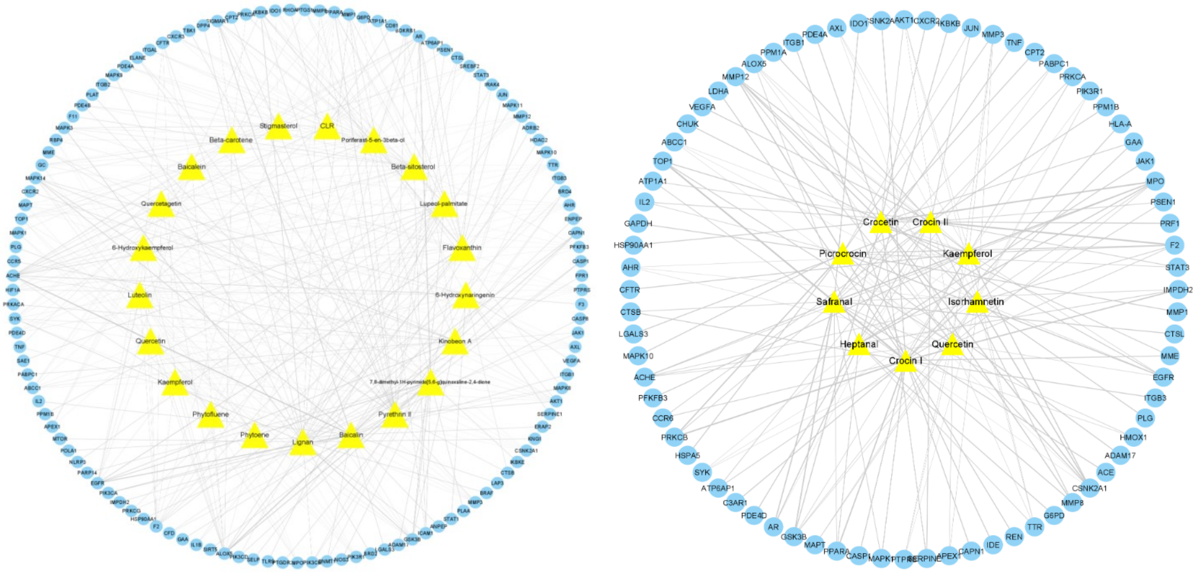

A B

Supplementary Figure 1. Network of Honghua (A) and Xihonghua (B) with active ingredients

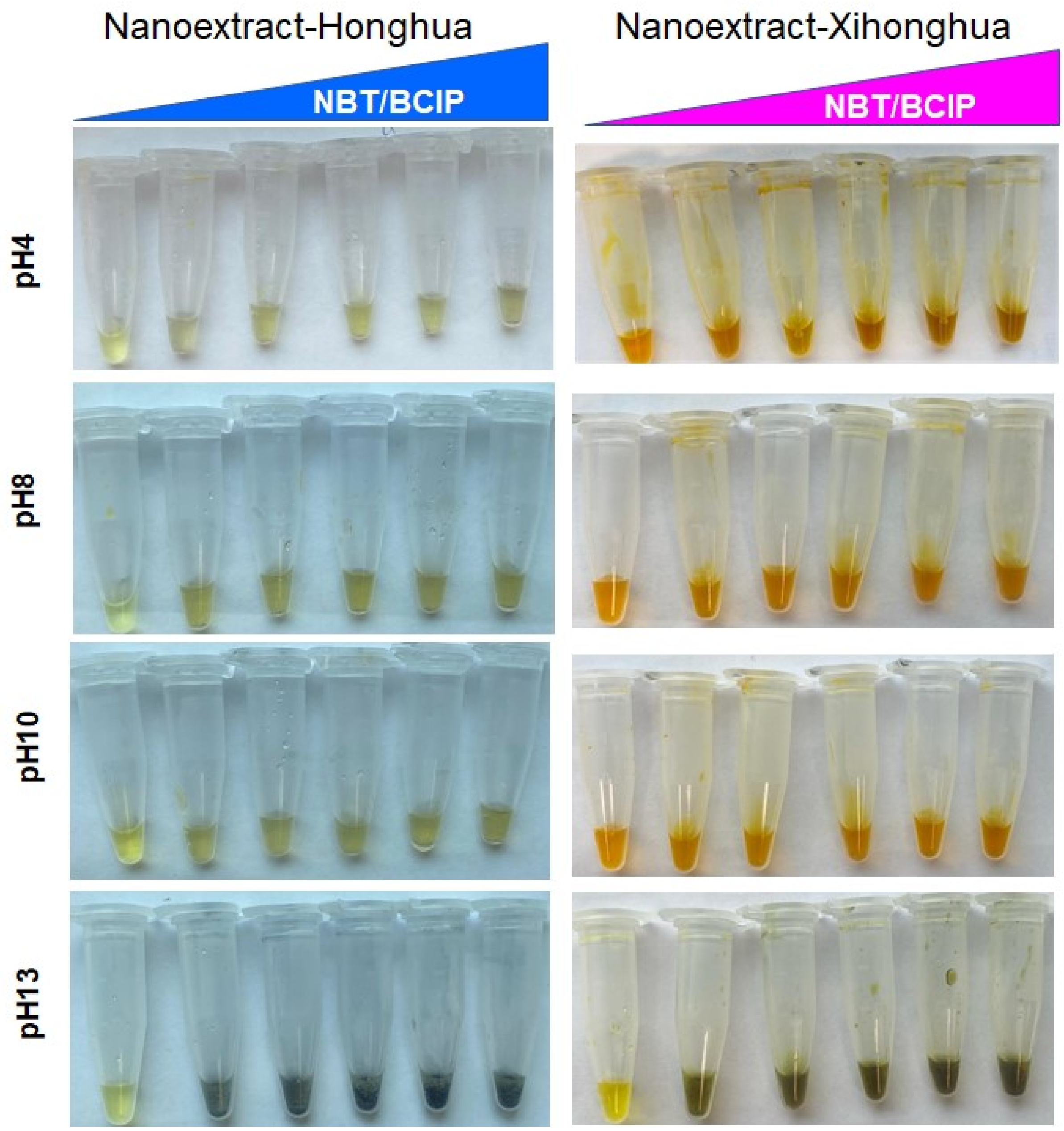

Supplementary Figure 2. The nanoscale of Honghua and Xihonghua extracts exhibit nanozyme activities at different pH. NBT/BCIP(0-,26-,32-,38-,42-,48-μL of commercial ready to use solution in 100 μL) concentration-dependent activity by pH effect is shown after 30 min reaction.
